# Prognostic Significance of Albumin in Modern Left Ventricular Assist Device Therapy: Relevance in the HeartMate 3 Era?

**DOI:** 10.3390/jcm14176193

**Published:** 2025-09-02

**Authors:** Roxana Moayedifar, Muhammed Celik, Barbara Karner, Anne-Kristin Schaefer, Hebe Al Asadi, Christiane Marko, Lukas Ruoff, Daniel Zimpfer, Julia Riebandt, Thomas Schlöglhofer

**Affiliations:** 1 Department of Cardiac and Aortic Thoracic Surgery, Medical University Vienna, 1090 Vienna, Austriathomas.schloeglhofer@meduniwien.ac.at (T.S.); 2 Center for Medical Physics and Biomedical Engineering, Medical University Vienna, 1090 Vienna, Austria; 3 Ludwig Boltzmann Institute for Cardiovascular Research, 1090 Vienna, Austria

**Keywords:** left ventricular assist device, mechanical circulatory support, HeartMate 3, biomarker, risk factor

## Abstract

**Background/Objectives**: Preoperative hypoalbuminemia is a known risk factor for adverse outcomes in cardiac surgery, but its role in patients undergoing HeartMate 3 (HM3) left ventricular assist device (LVAD) implantation is unclear. This study evaluated the association between albumin levels and postoperative outcomes, aiming to define a clinically meaningful cut-off for risk stratification. **Methods**: We retrospectively analyzed 205 adult patients who underwent HM3 implantation at a single center from June 2014 to December 2023. Receiver operating characteristic (ROC) analysis identified an optimal pre-implant albumin cut-off of <32 g/L. This threshold, derived using the maximal Youden Index, provided a sensitivity of 52.1%, specificity of 71.6%, and an AUC of 0.64 (95% CI 0.56–0.71), with internal bootstrapping validation confirming model stability, and calibration demonstrating good agreement between predicted and observed outcomes. Kaplan–Meier analysis assessed freedom from hemocompatibility-related adverse events (HRAEs) and survival. Cox proportional hazards models evaluated albumin and other variables as independent risk factors for HRAEs. **Results**: Patients with pre-implant albumin <32 g/L had higher rates of HRAEs, including stroke (24.9% vs. 8.4%, *p* = 0.004) and bleeding (38.1% vs. 23.2%, *p* = 0.012). Freedom from HRAEs was significantly lower in the hypoalbuminemia group (45.2% vs. 69.8%, *p* < 0.001) and competing risk-adjusted cumulative incidence for HRAE was higher, but did not reach statistical significance (*p* = 0.11), one-year HRAE-free survival was also reduced (68.5% vs. 85.7%, *p* = 0.03). In multivariable analysis, low albumin (HR 0.56, 95% CI 0.33–0.93, *p* = 0.026) and temporary right ventricular assist device (RVAD) support (HR 3.32, 95% CI 2.05–5.39, *p* < 0.001) were independent predictors of HRAEs. **Conclusions**: Low preoperative albumin is independently associated with increased HRAEs and reduced one-year survival after HM3 implantation. Compared with the traditional 35 g/L threshold, the ROC-derived 32 g/L cut-off offered superior balance between sensitivity and specificity, underscoring its clinical utility. Albumin may serve as a simple, pragmatic, and cost-effective biomarker for preoperative risk assessment and optimization.

## 1. Introduction

Left ventricular assist devices (LVADs) have become a cornerstone in the management of advanced heart failure, offering both bridge-to-transplant and destination therapy options [1]. Preoperative hypoalbuminemia has been identified as a predictor of increased mortality and postoperative complications in LVAD patients [2,3]. However, much of this research was conducted on earlier-generation devices, such as the HeartMate I and II (Abbott, Chicago, IL, USA), which were associated with higher rates of postoperative adverse events [4,5]. Since 2021, the HeartMate 3 (HM3; Abbott, Chicago, IL, USA) has been the only durable LVAD available on the market, consistently demonstrating superior outcomes, including lower rates of pump thrombosis, stroke, and right heart failure [1,4]. Despite these advancements, the impact of hypoalbuminemia on HM3 outcomes remains unclear.

Serum albumin is a recognized marker of nutritional status and systemic inflammation, widely studied in cardiac surgery and heart transplantation [6,7,8,9,10]. Low preoperative levels have been consistently linked to higher postoperative mortality and morbidity, reinforcing its role as a significant prognostic indicator in surgical outcomes [6,7,8]. While some evidence suggests similar trend in LVAD recipients, research is limited. Hypoalbuminemia in this population has been associated with acute kidney injury, infections, gastrointestinal bleeding, and neurological dysfunction, prolonged hospitalization, and increased healthcare utilization [3,11]. A 2014 study of 272 patients with HeartMate II (Abbott, Chicago, IL, USA) and HeartWare HVAD (Medtronic, Minneapolis, MN, USA) devices found significantly reduced survival at three and twelve months in those with low pre-operative albumin levels [2]. However, these devices are now obsolete, limiting the relevance of earlier findings.

Given the improved safety profile of the HM3 [1,4], the prognostic significance of pre-operative albumin in this context remains uncertain. This study aims to address this gap by evaluating the prognostic value of preoperative serum albumin in HM3 patients, providing insights that could help guide clinical decision-making and improve patient outcomes.

## 2. Materials and Methods

### 2.1. Study Design and Patient Population

**Figure 1:**
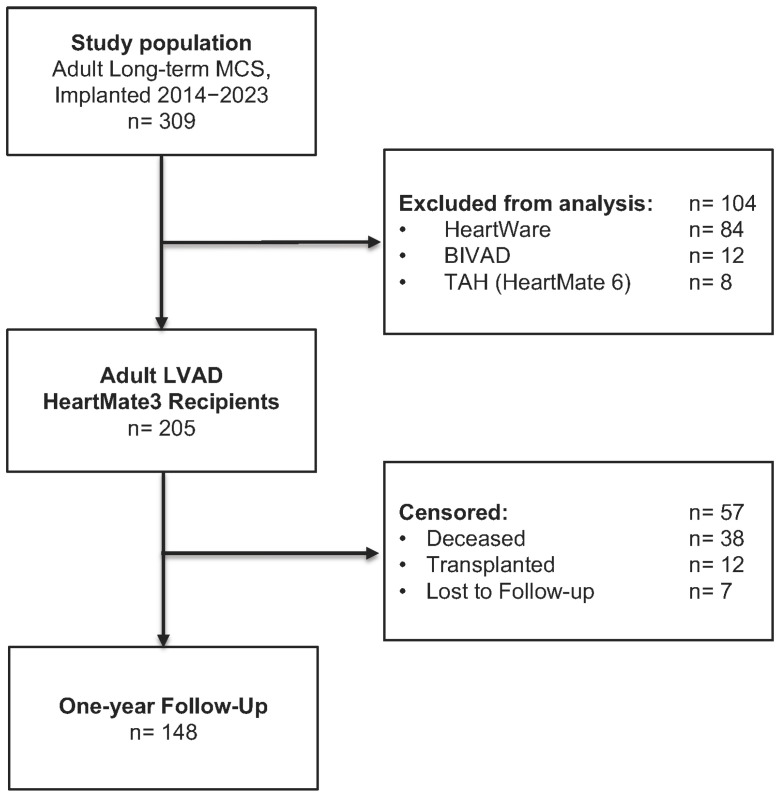
Study population; BIVAD = biventricular assist device; LVAD = left ventricular assist device; MCS = mechanical circulatory support; TAH = total artificial heart.

This retrospective, single-center cohort study included 205 consecutive adults who underwent HM3 (Abbott, Chicago, IL, USA) LVAD implantation for terminal heart failure between June 2014 and December 2023 (Figure 1). Patients receiving isolated right ventricular support, permanent biventricular assist devices (BIVAD), or a total artificial heart (HeartMate 6) were excluded. The study evaluated the prognostic value of preoperative serum albumin and aimed to identify a clinically meaningful hypoalbuminemia cut-off associated with HRAEs and survival.

Follow-up data, survival status, and lab parameters were obtained from the institutional VAD database (FileMaker Pro18) and patients’ digital health records. Data were collected during the index hospitalization or routine outpatient follow-up. Final survival status was assessed on 30 November 2024. The study was IRB-approved (EC Number: 1646/2024) with a waiver of consent.

### 2.2. Evaluation of Albumin Status

Serum albumin levels were measured at predefined time points to assess both preoperative status and postoperative trends. Measurements were obtained at our department using standard laboratory procedures and expressed in grams per liter (g/L), with a reference range of 35–52 g/L. Albumin was assessed preoperatively (within 48 h before LVAD implantation), and postoperatively on day 7, day 14, and at 1, 2, and 3 months following surgery. These time points were selected to evaluate early postoperative changes in albumin levels and their potential association with clinical outcomes.

### 2.3. Outcome Definition and Definition of Hemocompatibility-Related Adverse Events (HRAEs)

The primary outcome was the occurrence of HRAEs within one year post-HM3 implantation, stratified by albumin level. HRAEs were defined per previous HM3 trial criteria [4,5,12] and included bleeding, stroke, and pump thrombosis. Stroke was defined as any new, clinically evident neurological deficit of vascular origin, confirmed via imaging or clinical assessment, and categorized as ischemic or hemorrhagic. Bleeding included any event requiring surgical re-exploration, hospitalization for transfusion, or resulting in death. Events were stratified by timing, early (<24 h) and late (>24 h), and classified by location as surgical or gastrointestinal. Pump device malfunction, including pump thrombosis and outflow graft obstruction, encompassed any mechanical complication requiring surgical intervention, thrombolytics, or showing impaired function confirmed by clinical, imaging, or hemodynamic findings. These events were chosen for their significant impact on postoperative morbidity and mortality and analyzed relative to preoperative albumin.

Secondary outcomes included all-cause survival at 30 days and one year to distinguish early versus mid-term outcomes, along with t identification of independent perioperative risk factors for HRAEs.

### 2.4. Risk Factor Assessment

Preoperative serum albumin levels were evaluated as a potential independent risk factor for HRAEs and survival following HM3 implantation. The discriminative ability of preoperative albumin for predicting HRAE was assessed using receiver operating characteristic (ROC) analysis, with 95% confidence intervals (CIs) for the area under the curve (AUC) calculated according to the method of Hanley and McNeil [13]. The optimal cut-off was derived using the maximal Youden Index. Internal validation was performed using bootstrap resampling (5000 iterations), providing optimism-corrected estimates of model performance. Calibration was evaluated by comparing observed and expected event rates, expressed as the observed-to-expected (O/E) ratio.

Additional covariates included in the risk assessment model were: age, gender, body mass index (BMI) at implantation, INTERMACS profile at implantation, hypertension, peripheral artery disease, chronic kidney disease, diabetes type 2, cardiac infarction, atrial fibrillation, peripheral edema, stroke history, previous cardiac surgery, concomitant procedures, type of cardiopulmonary bypass support, less invasive surgical approach, site of distal anastomosis, the use of temporary right ventricular assist device (RVAD) support, nutritional scores, central venous pressure, mean pulmonary artery pressure and additional laboratory results pre-implantation (bilirubin, creatine, creatine kinase, GFR, white blood cell count, CRP, cholesterol, ALAT, sodium). Multivariable regression analysis was performed to determine the independent association between hypoalbuminemia and HRAEs, adjusting for these potential confounders.

### 2.5. Statistical Analysis

Continuous variables are presented as mean ± standard deviation or median (interquartile range), depending on normality assessed via the Kolmogorov–Smirnov test. Categorical variables are reported as counts (percentages). Group comparisons used the chi-square test for categorical variables and the independent samples *t*-test or Mann–Whitney U test for continuous variables, based on distribution.

The discriminative ability of preoperative albumin for predicting HRAE was assessed using ROC analysis, with 95% CIs for the AUC, and the optimal cut-off was derived using the maximal Youden Index.

Time-to-event outcomes were analyzed using competing-risk Fine–Gray models to assess the cumulative incidence of HRAEs, while HRAE-free survival was evaluated using Kaplan–Meier survival analysis and group differences tested using the log-rank test. Patients were censored at the time of heart transplantation, last follow-up, or LVAD explantation due to recovery.

Univariable Cox regression was first applied to identify potential predictors of HRAEs. Variables with *p* < 0.05 were subsequently entered into a multivariable Cox proportional hazards model. The proportional hazards assumption was formally tested both visually, using scaled Schoenfeld residuals, and statistically with chi-square global tests. As no violation was observed, time-dependent terms or stratification were not required. Results are reported as hazard ratios (HRs) with 95% CIs.

All statistical analyses were conducted using IBM SPSS Statistics Version 28.0 (IBM Corp., Armonk, NY, USA) and Stata/SE 29.0 (StataCorp LLC, College Station, TX, USA). Statistical significance was defined as a two-sided *p*-value < 0.05.

## 3. Results

### 3.1. Patient Demographics and Baseline Characteristics

Preoperative characteristics and comorbidities of the overall study population (*n* = 205), as well as those stratified by preoperative albumin levels, low albumin (<32 g/L, *n* = 119) and normal albumin (≥32 g/L, *n* = 86), are summarized in Table 1.

However, differences emerged in preoperative clinical status and intraoperative management. A significantly higher proportion of patients in the hypoalbuminemia group were classified as INTERMACS profile 1 at the time of implantation (31.9% vs. 7.0%, *p* < 0.001), indicating more severe hemodynamic compromise. Regarding surgical approach, a less invasive implantation strategy was more frequently used in the normal-albumin group (43.0% vs. 26.1%, *p* = 0.016). Additionally, patients in the low-albumin group required RVAD support more frequently (43.1% vs. 23.3%, *p* = 0.003), suggesting a higher incidence of perioperative right heart failure in this cohort.

### 3.2. Albumin Cut-Off Determination

**Figure 2:**
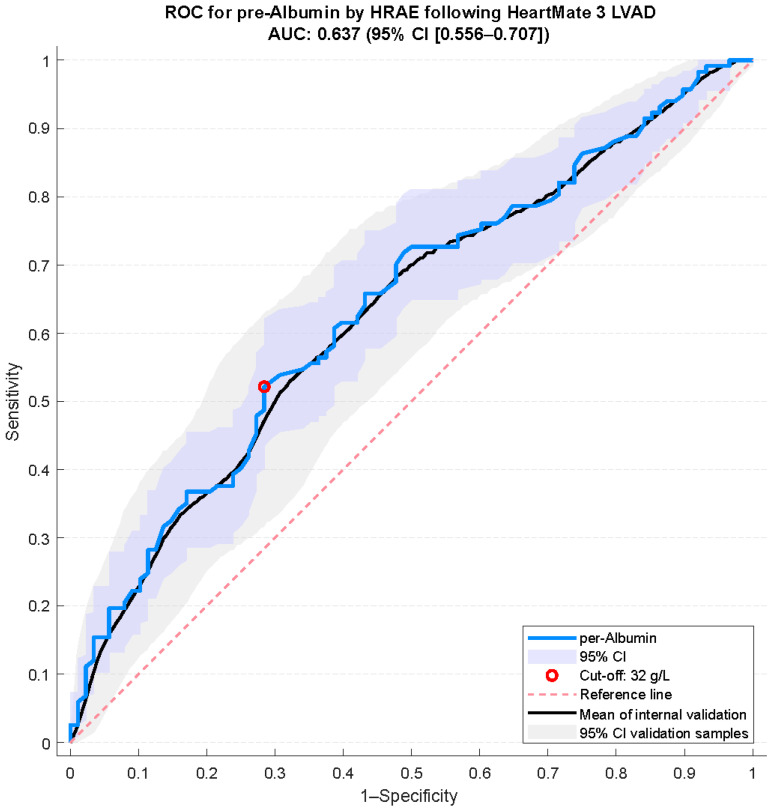
Receiver operating characteristic (ROC) analysis for preoperative albumin predicting hemocompatibility-related adverse events (HRAEs) following HeartMate 3 LVAD implantation including internal validation using bootstrap resampling (5000 iterations), providing optimism-corrected estimates of model performance. AUC = area under the curve.

To identify a clinically meaningful threshold for preoperative hypoalbuminemia in predicting HRAEs after HM3 implantation, an ROC analysis was conducted. The optimal cut-off value was identified as 32 g/L, yielding a sensitivity of 52.1%, a specificity of 71.6%**,** and an AUC of 0.64 (95% CI 0.56–0.71) (Figure 2). These values indicate a modest ability of preoperative albumin levels to discriminate between patients who did and did not experience HRAEs within the first postoperative year (Figure 2). Internal validation demonstrated consistent performance with narrow confidence intervals, underscoring the robustness of the cut-off. Calibration O/E ratio resulted in 1.048 (88 observed HRAEs/84 predicted HRAEs) revealing a slight overestimation.

### 3.3. Hemocompatibility-Related Adverse Events

**Figure 3:**
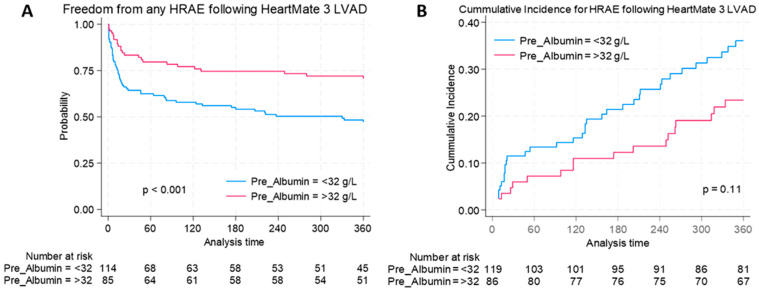
Hemocompatibility-related adverse events (HRAEs) by preoperative albumin status in HeartMate 3 LVAD recipients: (**A**) Freedom from any HRAE one year following implantation stratified by pre-operative albumin level; (**B**) cumulative incidence of any HRAE by preoperative albumin level (Fine–Gray competing-risks analysis).

Following identification of the optimal cut-off value at 32 g/L, patients were stratified into low albumin (<32 g/L) and normal albumin (≥32 g/L) cohorts for further analysis. Using this threshold, unadjusted freedom from any HRAE was significantly higher in the normal albumin cohort (69.8%) compared to the lower albumin cohort (45.2%, *p* < 0.001; Figure 3A). In line with this, the cumulative incidence of HRAEs was higher among patients with low albumin, although this did not reach statistical significance (*p* = 0.11; Figure 3B).

Freedom from any stroke was also significantly greater in the normal albumin group (91.6% vs. 75.1%, *p* = 0.004). When analyzed by stroke subtype, freedom from ischemic stroke did not reach statistical significance (95.0% vs. 87.5%, *p* = 0.063), while freedom from hemorrhagic stroke was significantly higher in patients with normal pre-operative albumin levels (96.4% vs. 83.5%, *p* = 0.008). Freedom from any bleeding was significantly higher in the normal albumin cohort (76.8% vs. 61.9%, *p* = 0.012), as was freedom from surgical bleeding (88.2% vs. 75.2%, *p* = 0.020). There was no significant difference in freedom from gastrointestinal bleeding between the groups (88.5% vs. 81.1%, *p* = 0.130).

### 3.4. Survival Outcomes

**Figure 4:**
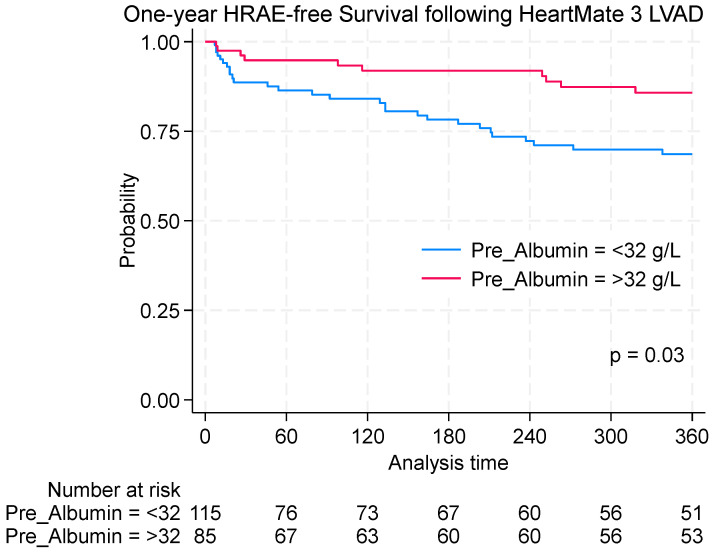
Kaplan–Meier analysis of one-year outcomes following HeartMate 3 LVAD implantation stratified by preoperative albumin levels. One-year HRAE-free survival in patients with hypoalbuminemia (<32 g/L) compared with those with normal albumin levels (≥32 g/L).

There was no statistically significant difference in 30-day survival between the two cohorts, with survival rates of 95.3% in patients with normal albumin levels and 90.8% in those with hypoalbuminemia (*p* = 0.210). However, one-year outcomes differed significantly. Patients with normal albumin levels (≥32 g/L) had a one-year HRAE-free survival of 85.7% compared with 68.5% in the hypoalbuminemia group (<32 g/L; log-rank *p* = 0.03; Figure 4). These findings suggest that preoperative hypoalbuminemia is associated with impaired event-free survival following HM3 LVAD implantation.

### 3.5. HRAEs: Risk Factor Analysis

A total of 33 variables, as presented in Table 1, were included in the univariate Cox proportional hazard analysis to assess their association with the occurrence of HRAEs within the first year following HM3 implantation. The univariate analysis (Table A1) showed that four variables were significantly associated with HRAE risk. Chronic kidney disease was associated with an increased risk (HR 1.63, 95% CI: 1.02–2.59, *p* = 0.040), while higher preoperative serum albumin levels were protective (HR 0.43, 95% CI: 0.26–0.71, *p* < 0.001). Additionally, less invasive surgical HM3 implantation approaches were associated with a reduced risk for HRAEs (HR 0.58, 95% CI: 0.34–0.97, *p* = 0.038), whereas the need for RVAD support significantly increased the risk (HR 3.91, 95% CI: 2.47–6.16, *p* < 0.001).

**Figure 5:**
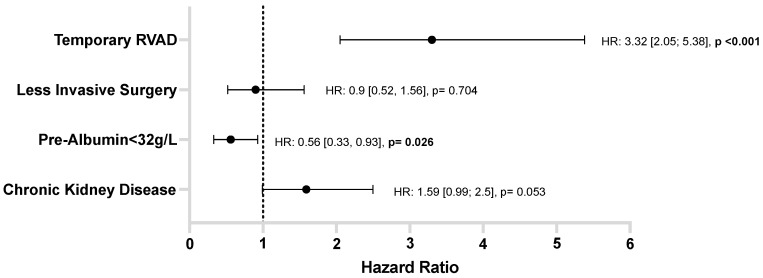
Forest plot of the multivariable cox proportional hazards analysis for risk of hemocompatibility-related adverse events within one year post–LVAD implantation. HR = hazard ratio; RVAD = right ventricular assist device.

In the multivariable Cox regression model, preoperative albumin level remained an independent predictor of HRAEs (HR 0.56, 95% CI: 0.33–0.93, *p* = 0.026), along with temporary RVAD support, which was also independently associated with an increased risk (HR 3.32, 95% CI: 2.05–5.38, *p* = 0.001; Figure 5). Schoenfeld residuals showed no deviations over time, and the chi-square global covariate test (χ^2^ = 6.96, df = 4, *p* = 0.137) was non-significant, indicating the proportional hazards assumption was met.

### 3.6. Longitudinal Changes in Albumin

**Figure 6:**
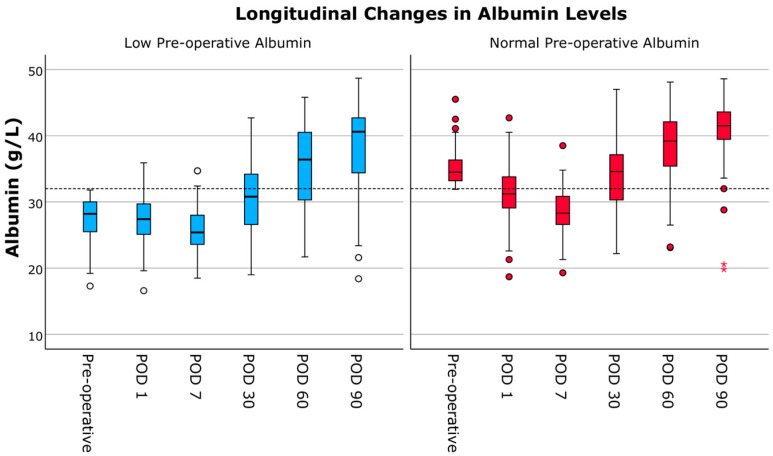
Longitudinal changes in albumin levels stratified by pre-operative albumin (low albumin < 32 g/L and normal albumin ≥ 32 g/L). Dashed line represents 32 g/L cut-off. POD = post-operative day. ** indicating outliers.

Pre-operative albumin levels were significantly lower in the low-albumin group compared to the normal-albumin group (28.1 (4.3) g/L vs. 34.7 (3.8) g/L, *p* < 0.001), and remained significantly lower at all longitudinal follow-up assessments (*p* < 0.006; Figure 6). In the hypoalbuminemia group, albumin levels were below the 32 g/L cut-off at all time points except months 2 and 3. In contrast, in the normal group, only the values on postoperative days 1 and 7 fell below the cut-off.

## 4. Discussion

Hypoalbuminemia has been widely recognized as a marker of increased morbidity and mortality across cardiac surgical populations. Low preoperative albumin levels have been associated with a higher rate of infections, bleeding, acute kidney injury, and respiratory failure, as well as prolonged intensive care unit (ICU) and hospital stays [3,7,14,15,16,17]. In this retrospective, single-center cohort study of 205 HM3 LVAD recipients, lower preoperative serum albumin (<32 g/L) was significantly associated with a higher incidence of HRAEs and reduced one-year survival. Alongside albumin, temporary RVAD support also emerged as an independent predictor of adverse outcomes, reflecting the higher baseline severity and hemodynamic instability of these patients. While low albumin similarly remained independently associated with risk, the two variables likely capture distinct but complementary aspects of patient vulnerability, nutritional/inflammatory reserve on the one hand, and perioperative circulatory instability on the other. This emphasizes that albumin is not merely a surrogate for severity but provides additive prognostic information beyond established clinical risk factors.

Our findings align with prior studies linking hypoalbuminemia to increased morbidity and mortality in cardiac surgery [3,7,14,15,16,17]. In coronary artery bypass grafting (CABG) patients, low albumin has been associated with poor long-term survival, despite minimal impact on early mortality [7]. In the off-pump coronary artery bypass (OPCAB) procedures, it correlates with increased respiratory failure, wound complications, and inotropic requirements [17]. Likewise, a study of 822 heart transplant recipients showed low preoperative albumin predicted reduced one-year survival, emphasizing its prognostic value in advanced cardiac therapies [6]. These parallels strengthen the evidence that albumin is not only a biomarker of nutritional and inflammatory status but also a meaningful determinant of outcomes in patients undergoing durable MCS.

Although data on LVAD recipients remain relatively limited, prior studies have consistently shown that lower preoperative albumin levels predict a broad range of adverse outcomes [3,18]. These include increased rates of acute renal failure, infections, gastrointestinal bleeding, neurological dysfunction, and prolonged hospitalization [3,11]. Earlier work in first- and second generation LVAD populations suggested an association with prolonged ICU stay, renal dysfunction, and sepsis [3,19], with variable impact on survival. More recent studies, however, have demonstrated significantly reduced survival in patients with hypoalbuminemia at both 3 and 12 months, and up to 2 years post-implantation [2,11,20]. In line with these findings, our data show that hypoalbuminemia not only predicts morbidity but also correlates with reduced event-free survival, highlighting its dual impact on both quality and quantity of survival in HM3 recipients.

Notably, normalization of albumin levels following implantation has been associated with improved outcomes, suggesting a potentially modifiable trajectory [2]. Furthermore, perioperative increases in albumin levels are predictive of reduced morbidity and mortality, emphasizing the dynamic prognostic role of albumin [19,21]. Tools such as the MELD-XI score, which incorporates albumin, have demonstrated strong predictive value for 1-year survival in LVAD recipients [20]. In our cohort, preoperative albumin levels were significantly lower in the hypoalbuminemia group and remained below the 32 g/L threshold up to month 2, whereas patients in the normal-albumin group recovered above this threshold after postoperative day 7, supporting the prognostic relevance of both baseline status and longitudinal trends.

Importantly, compared with the traditional 35 g/L threshold, the 32 g/L cutoff offered a more balanced predictive profile, with markedly higher sensitivity and overall discrimination, making it clinically more useful for identifying high-risk patients. While the discriminative ability of preoperative albumin was modest, the identified threshold of 32 g/L provided the best balance between sensitivity and specificity for risk stratification in this cohort. The identification of an optimal preoperative albumin threshold was a central aspect of our analysis. While the traditionally used cutoff of 35 g/L [3,7] has been applied in previous studies, our data-driven approach identified 32 g/L as a more suitable value for risk stratification in this cohort. To ensure robustness and avoid potential overfitting, we performed internal validation using bootstrapping techniques, which confirmed the stability of the derived threshold. In addition, calibration O/E ratio was assessed and demonstrated good agreement between predicted and observed outcomes, supporting the validity of the model. When directly compared to the conventional threshold, the 32 g/L cutoff provided a superior balance between sensitivity (52% vs. 24%) and specificity (72% vs. 90%), as reflected by a higher Youden Index (0.237 vs. 0.137). This improvement in discriminatory ability enhances clinical applicability, as it increases both the identification of high-risk patients (positive predictive value 58%) and the exclusion of low-risk patients (negative predictive value 67%). Taken together, these findings suggest that the 32 g/L cutoff may provide a more accurate and clinically meaningful risk marker than the traditional 35 g/L threshold, while still requiring external validation in larger, multi-center cohorts.

Our findings reinforce the clinical relevance of preoperative albumin as a simple and cost-effective biomarker for identifying patients at increased risk of adverse outcomes following HM3 implantation. As a marker reflecting both nutritional status and systemic inflammation, albumin provides valuable insight into the patient’s physiological reserve and vulnerability to complications [11]. Establishing a context-specific cut-off adds granularity and enhances the precision of risk stratification, potentially informing earlier intervention and individualized management. Notably, while albumin was included in the HeartMate II risk score [22], it is not currently incorporated in the HM3 risk score [23], despite its demonstrated association with morbidity and mortality in this and other studies. Incorporating albumin into future risk models could improve prognostic accuracy and guide clinical decision-making. This concept is further supported by the Prognostic Nutritional Index (PNI), which incorporates serum albumin and lymphocyte count to reflect nutritional status [24]. Studies have shown that a low PNI (<30) in LVAD patients is associated with longer postoperative hospital stays, increased rates of right heart failure, acute renal failure, and higher mortality [24,25]. PNI has also emerged as an independent predictor of survival and indicator of the need for device replacement [26,27], but did not serve as an independent predictor for HRAEs in this study. Other indices such as the Nutritional Risk Index (NRI) and CONUT score not only provide prognostic value, but also collectively highlight the critical role of nutrition in LVAD outcomes [28,29,30]. However, in our study, neither PNI nor CONUT added predictive value beyond albumin and neither served as an independent predictor for HRAEs, underlining that albumin alone offered the strongest and most clinically meaningful prognostic signal in this cohort. Comprehensive preoperative nutritional assessment and intervention using multidisciplinary approaches should be considered as part of routine patient optimization.

## 5. Limitations

Study strengths include the uniform use of the HM3 device, robust single-center data, and consistent one-year follow-up. Our ROC-derived albumin cut-off adds precision beyond traditional thresholds. However, the retrospective design and single-center setting introduce potential residual confounding and limit causal inference, as unmeasured factors and center-specific practices may influence outcomes. The single-center nature also limits generalizability, particularly given the predominantly male patient population (~91%). Furthermore, patients in the low-albumin group presented with greater baseline severity, as reflected by higher proportions of INTERMACS 1–2 profiles and increased need for temporary RVAD support, which may have contributed to worse outcomes independent of albumin levels.

We intentionally excluded patients receiving other durable LVAD types or total artificial hearts (TAHs) to ensure a homogeneous HM3 cohort, as device type is a strong confounder for adverse event rates. While this approach improved internal validity, it may limit generalizability and could have led to an underestimation of HRAE rates compared with more heterogeneous real-world populations.

Albumin alone demonstrated only modest discriminative power for predicting HRAEs, with an AUC of 0.64 (95% CI 0.56–0.71), sensitivity of 52.1%, and specificity of 71.6%. While we did capture serial albumin measurements, the interpretation of longitudinal changes remains limited, and standardized approaches for reporting perioperative biomarker trends are lacking. Clearer guidelines on the assessment and reporting of temporal albumin dynamics are needed to promote transparency and ensure comparability across studies. We also lacked data on certain potentially relevant baseline parameters, such as echocardiographic baseline measurements and preoperative albumin-targeted interventions. Importantly, the lack of statistical significance for some associations likely reflects the limited sample size and event rate in our cohort, rather than the absence of a true biological effect. Nonetheless, internal validation by bootstrapping confirmed the stability of this finding, and calibration O/E ratio showed acceptable agreement between observed and predicted HRAEs. Still, these results highlight that albumin should be viewed as a complementary biomarker rather than a stand-alone predictor. Moreover, although we explored composite nutritional–inflammatory indices (PNI, CONUT) and inflammatory markers (CRP, WBC), they did not provide additional predictive value beyond albumin, consistent with its strong correlation with PNI. This underlines the utility of albumin as a pragmatic biomarker, while also reflecting the need for more comprehensive multimodal risk stratification.

Finally, while our results suggest that persistently lower albumin levels are associated with both higher HRAE incidence and reduced one-year survival, causality cannot be established, and the potential impact of albumin-targeted interventions remains unknown. Prospective, multi-center studies with systematic baseline nutritional and inflammatory assessment are warranted to validate these findings, confirm the prognostic utility of the 32 g/L threshold, and determine whether albumin optimization can improve outcomes in LVAD recipients.

## 6. Conclusions

In conclusion, low preoperative serum albumin (<32 g/L) was independently associated with a higher incidence of HRAEs and reduced one-year HRAE free survival in HM3 LVAD recipients. Importantly, this ROC-derived cut-off provided a more clinically meaningful threshold than the traditional 35 g/L, with internal validation confirming its stability. Alongside temporary RVAD support, which also emerged as an independent predictor of adverse outcomes, albumin captures a distinct and complementary dimension of patient vulnerability, reflecting nutritional and inflammatory reserve. With the HM3 as the only durable LVAD currently in use, these findings have contemporary relevance and highlight albumin as a pragmatic, low-cost biomarker that could enhance current risk stratification models and guide patient optimization strategies in the modern era of mechanical circulatory support.

## Figures and Tables

**Table 1:** Baseline characteristics and implant data of the study population stratified by pre-operative albumin levels.

Variable *n* (%), Mean ± SD or Median (IQR)	Albumin < 32 g/L *N* = 119	Albumin ≥ 32 g/L *N* = 86	*p*-Value
Patient characteristics			
Age (years)	63 ± 10.6	61 ± 8.8	0.905
Gender			0.465
male	106 (89.1)	80 (93.0)	
female	13 (10.9)	6 (7.0)	
BMI (kg/m^2^)	26.8 ± 4.8	27.8 ± 4.6	0.365
Cardiomyopathy			0.269
Dilated	47 (39.5)	27 (31.4)	
Ischemic	70 (58.8)	55 (64.0)	
Other	2 (1.6)	4 (4.6)	
INTERMACS			<0.001
Level 1	38 (31.9)	6 (7.0)	
Level 2	25 (21.0)	15 (17.4)	
Level 3	19 (16.0)	23 (26.7)	
Level 4–7	37 (31.1)	42 (48.8)	
Hypertension	38 (32.5)	29 (33.7)	0.881
Peripheral artery disease	7 (6.0)	4 (4.7)	0.763
Chronic kidney disease	35 (30.2)	24 (27.9)	0.756
Diabetes Type 2	38 (32.8)	32 (38.1)	0.605
Atrial fibrillation	53 (49.1)	34 (40.0)	0.244
Stroke history	12 (10.1)	5 (5.8)	0.320
Peripheral edema	22 (19.1)	17 (20.2)	0.860
Cardiac infarction	36 (30.3)	27 (31.4)	0.880
Surgical implant data			
Previous cardiac surgery	14 (11.8)	10 (11.6)	1.000
Less invasive implant method	31 (26.1)	37 (43.0)	0.016
Bypass support			0.130
CPB	100 (84.9)	77 (89.5)	
ECMO	11 (9.2)	1 (1.2)	
Off-pump	6 (6.7)	7 (8.1)	
LVAD anastomosis site			0.845
Ascending aorta	112 (94.1)	82 (95.3)	
Descending aorta	1 (0.8)	1 (1.2)	
Left subclavian artery	6 (5.0)	3 (3.5)	
Temporary RVAD	52 (43.7)	20 (23.3)	0.003
Concomitant procedures	48 (40.3)	30 (34.9)	0.468
Pre-operative laboratory parameters			
Creatine kinase (U/L)	62.0 (59.0)	66.0 (111)	0.921
Creatine (mg/dL)	1.35 (0.74)	1.29 (0.96)	0.994
MDRD-GFR (ml/min)	57.8 (35.3)	56.4 (44.9)	0.941
Cholesterol (mg/dL)	122.4 ± 36.8	116.7 ± 49.5	0.941
Sodium (mmol/L)	138.9 ± 4.5	138.5 ± 5.4	0.618
ALAT (U/L)	29.0 (31.0)	40.5 (103.5)	0.002
Bilirubin (mg/dL)	0.92 (0.86)	1.09 (1.33)	0.286
WBC (G/L)	8.0 (4.0)	8.4 (4.5)	0.402
CRP (mg/dL)	1.3 (4.2)	2.8 (5.0)	0.197
Nutritional Scores			
PNI	39.1 (7.6)	39.2 (11.0)	0.598
CONUT	5 (4)	6 (4)	0.377
Pre-operative hemodynamics			
Central venous pressure (mmHg)	14.5 (9.0)	15.0 (7.0)	0.377
Mean pulmonary artery pressure (mmHg)	32.9 ± 11.4	33.1 ± 10.5	0.922

BMI = body mass index; CPB = cardiopulmonary bypass; ECMO = extracorporeal membrane oxygenation; RVAD = right ventricular assist device; MDRD-GFR = modification of diet in renal disease glomerular filtration rate; ALAT = Alanine aminotransferase; WBC = white blood count; CRP = C-reactive protein; PNI = prognostic nutritional index; CONUT = controlling nutritional status.

## Data Availability

The data presented in this study are available on request from the corresponding author.

## Abbreviations

The following abbreviations are used in this manuscript:

ALAT	Alanin-aminotransferase
AUC	Area under the curve
BIVAD	Biventricular assist device
BMI	Body mass index
CABG	Coronary artery bypass grafting
CI	Confidence interval
CONUT	Controlling nutritional status
CRP	C-reactive protein
HM3	HeartMate 3
HR	Hazard ratio
HRAE	Hemocompatibility-related adverse events
ICU	Intensive care unit
LVAD	Left ventricular assist device
MDRD-GFR	Modification of diet in renal disease glomerular filtration rate
NRI	Nutritional risk index
OPCB	Off-pump coronary artery bypass
PNI	Prognostic nutritional index
ROC	Receiver operating characteristic
RVAD	Right ventricular assist device
VAD	Ventricular assist device
WBC	White blood count

## Appendix A

**Table A1:** Univariate analysis of variables associated with risk of hemocompatibility-related adverse events (HRAEs).

Variable	HR	95% CI	*p*-Value
Age (years)	1.00	[0.98; 1.03]	0.930
Gender (female vs. male)	1.03	[0.47; 2.23]	0.946
BMI	0.97	[0.93; 1.02]	0.266
INTERMACS			
Level 1 (Reference)	1.00	-	-
Level 2	0.82	[0.43; 1.57]	0.547
Level 3	0.58	[0.29; 1.15]	0.120
Level 4–7	0.56	[0.32; 1.01]	0.055
Hypertension	0.66	[0.40; 1.10]	0.111
Peripheral artery disease	0.92	[0.34; 2.51]	0.868
Chronic kidney disease	1.63	[1.02; 2.59]	0.04
Diabetes type 2	1.00	[1.00; 1.00]	0.225
Cardiac infarction	1.01	[0.64; 1.59]	0.954
Atrial fibrillation	1.43	[0.93; 2.21]	0.103
Peripheral edema	1.55	[0.95; 2.54]	0.080
Stroke history	1.72	[0.89; 3.33]	0.107
Serum-albumin (pre-operative)			
<32 g/L	1.00	-	-
≥32 g/L	0.43	[0.26; 0.71]	<0.001
Previous cardiac surgery	1.17	[0.60; 2.28]	0.638
Concomitant procedures	0.92	[0.59; 1.41]	0.692
Bypass support type			
Off-pump vs. CPB (Reference)	0.89	[0.36; 2.20]	0.792
ECMO vs. CPB (Reference)	1.43	[0.62; 3.31]	0.398
Less invasive implant method	0.58	[0.34; 0.97]	0.038
LVAD anastomosis site			
Ascending Aorta (Reference)	1.00	-	-
Other (Subclavian/Descending Aorta)	2.03	[0.93; 4.42]	0.074
Temporary RVAD support	3.91	[2.47; 6.16]	<0.001
PNI	1.00	[1.00; 1.00]	0.186
CONUT Score	0.96	[0.84; 1.09]	0.522
CONUT categorial			
Normal (Reference) vs. Mild	1.04	[0.14; 7.86]	0.972
Normal (Reference) vs. Moderate	0.59	[0.08; 4.50]	0.614
Normal (Reference) vs. Severe	0.91	[0.11; 7.30]	0.931
Central venous pressure (mmHg)	1.01	[0.97; 1.04]	0.735
Mean pulmonary artery pressure (mmHg)	0.99	[0.97; 1.02]	0.553
Bilirubin (mg/dL)	0.98	[0.86; 1.12]	0.742
Creatine (mg/dL)	1.07	[0.84; 1.35]	0.586
MDRD-GFR (mL/min)	0.99	[0.99; 1.00]	0.066
WBC (G/L)	1.02	[0.98; 1.07]	0.356
CRP (mg/dL)	1.00	[0.97; 1.04]	0.878
Cholesterol (mg/dL)	1.00	[0.99; 1.01]	0.780
Creatine kinase (U/L)	1.00	[1.00; 1.00]	0.590
ALAT (U/L)	1.00	[1.00; 1.00]	0.304
Sodium (mmol/L)	1.00	[0.96; 1.05]	0.902

BMI = body mass index; CI = confidence interval; CPB = cardiopulmonary bypass; ECMO = extracorporeal membrane oxygenation; HR = hazard ratio; LVAD = left ventricular assist device; RVAD = right ventricular assist device; MDRD-GFR = modification of diet in renal disease glomerular filtration rate; ALAT = Alanine aminotransferase; WBC = white blood count; CRP = C-reactive protein; PNI = prognostic nutritional index; CONUT = controlling nutritional status.
